# Knockout of calpain‐1 protects against high‐fat diet‐induced liver dysfunction in mouse through inhibiting oxidative stress and inflammation

**DOI:** 10.1002/fsn3.2002

**Published:** 2020-12-22

**Authors:** Hao Xu, Li Zhang, Duowen Xu, Weibo Deng, Wenbao Yang, Futian Tang, Mingxu Da

**Affiliations:** ^1^ Department of Oncology Surgery Gansu Provincial People's Hospital Lanzhou Gansu Province China; ^2^ School of Clinical Medicine Ningxia Medical University Yinchuan Ningxia Hui Autonomous Region China; ^3^ Pharmacy Department Shaanxi Aerospace Hospital Xi'an Shaanxi Province China; ^4^ Pharmacy Department Wuwei Medical Academy Wuwei Gansu Province China; ^5^ School of Clinical Medicine Gansu University of Traditional Chinese Medicine Lanzhou Gansu Province China; ^6^ Key Laboratory of Digestive System Tumor of Gansu Province and Department of Cardiovascular Diseases Lanzhou University Second Hospital Lanzou Gansu Province China

**Keywords:** calpain‐1 knockout, hyperlipidemia, inflammation, liver dysfunction, oxidative stress

## Abstract

The present study was designed to investigate the significance of calpain‐1 in the high‐fat diet (HFD)‐induced liver dysfunction and to explore the possible mechanism. C57 mice and calpain‐1 knockout (KO) mice were fed with standard diet (*SD*) or HFD, respectively, for 16 weeks. The activities of calpain, aspartate aminotransferase (AST), alanine aminotransferase (ALT), and superoxide dismutase (SOD) in serum and/or liver of mouse were measured. Lipid profiles in the serum and liver were examined. Contents of oxidized low‐density lipoprotein (oxLDL), malondialdehyde (MDA), tumor necrosis factor (TNF‐α), and interleukin‐6 (IL‐6) in serum or/and liver were detected. The results showed that compared with C57 mice fed with *SD*, HFD‐fed C57 mice showed the increased activities of AST and ALT in the serum, which was decreased in calpain‐1 KO mice fed with HFD. In addition, knockout of calpain‐1 decreased the contents of oxLDL, MDA, TNF‐α, and IL‐6, while increased SOD activity, in serum and/or liver. However, knockout of calpain‐1 had no effects on lipid profiles in both serum and liver. When fed with *SD*, all these parameters of C57 and calpain‐1 KO mice were comparable except for decreased calpain activity in the liver of calpain‐1 KO mice. The results suggested that knockout of calpain‐1 protects against HFD‐induced liver dysfunction through inhibiting oxidative stress and inflammation.

## Graphical abstract

Knockout of calpain‐1 attenuates hyperlipidemia‐induced liver dysfunction of mice. Knockout of calpain‐1 decreases contents of oxidized low‐density lipoprotein and increased activity of superoxide dismutase. Knockout of calpain‐1 decreases tumor necrosis factor and interleukin‐6 contents

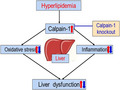

## 1 INTRODUCTION

Nonalcoholic fatty liver disease (NAFLD) is an epidemic global health problem, with a prevalence close to 30% in the general population, in parallel with obesity epidemic and occidental alimentary diet habits (Abdallah et al., 2020; Cho et al., 2014). NAFLD is a spectrum of liver diseases, since simple steatosis until cirrhosis and hepatocellular carcinoma. The progressive form called steatohepatitis, characterized by inflammation, progresses through fibrosis and liver structural changes (Guo et al., 2019).

The presence of hyperlipidemia (hypercholesterolemia, hypertriglyceridemia, or both) was identified in 20%–80% of NAFLD cases (Han et al., 2019). Inflammation is implied in the pathogenesis of NAFLD (Guo et al., 2019; Jiang et al., 2018; Kalantar et al., 2019). Injury of hyperlipidemia‐induced accumulation of visceral fat to the liver, the largest solid organ in the body, leads to a cascade of inflammatory events (Guo et al., 2019). Chronic inflammation leads to the activation of hepatic stellate cells that undergo transdifferentiation to become myofibroblasts, the main extracellular matrix producing cells in the liver. Accordingly, the mechanisms by which liver inflammation and fibrosis develop in chronic liver diseases are explored to identify appropriate and meaningful diagnostic targets for clinical practice. There is an increase in inflammatory markers such as IL‐6 and TNF‐α under hyperlipidemic condition, implying the involvement of inflammation in hyperlipidemia‐induced liver dysfunction (Guo et al., 2019).

There is inadequate information about the mechanisms of hyperlipidemia‐mediated liver dysfunction. As indicated in several studies, the formation of reactive oxygen species (ROS), besides nitric oxide (NO) synthesis and lipoperoxidation, increases in the liver as a result of high‐fat diet (HFD) administration. Conversely, hyperlipidemia results in the depletion of protective antioxidants, such as glutathione (GSH), and inhibits free radical scavenging enzymes, including catalase (CAT), superoxide dismutase (SOD), and glutathione peroxidase (GPx) (Kang et al., 2014; Kohli et al., 1997).

Calpain‐1, a Ca^2+^‐dependent cysteine protease, is implicated in a few of pathological conditions such as liver dysfunction associated with NAFLD (Kurikawa et al., 2013; Lazo & Clark, 2008). It has been previously reported that calpain‐1 is upregulated in the liver tissue of mice fed with HFD, highlighting the important role of calpain‐1 in the hyperlipidemia‐induced liver dysfunction (Lee et al., 2019). The involvement of calpain‐1 in liver dysfunction largely depends on its mediation of oxidative stress and inflammation, which are the most important contributors to the onset and progression of liver dysfunction (Li & Lu, 2018). In addition, a previous study has revealed that the inhibition of oxidative stress improves liver dysfunction induced by hyperlipidemia (Li et al., 2020). Furthermore, calpain‐1 was reported to mediate varieties of liver injury and inhibition of calpain‐1 activity and/or expression might protect the liver injury. Indeed, overexpression of homeodomain‐interacting protein kinase 2 (HIPK2) attenuates sepsis‐mediated liver injury by reducing the activity of calpain‐1 (Limaye et al., 2003). Impairment of autophagosome–lysosome fusion contributes to chronic ethanol‐induced liver injury by increasing the protein expression of calpain‐1 (Mehendale & Limaye, 2005). Melatonin inhibits mTOR‐dependent autophagy during liver ischemia/reperfusion (I/R) by decreasing the expression of calpain‐1 (Mei et al., 2015).

The present study was designed to investigate the role of calpain‐1 in hyperlipidemia‐induced liver dysfunction by using calpain‐1 knockout mouse fed with HFD. The results indicated that knockout of calpain‐1 protected against the hyperlipidemia‐induced liver dysfunction through inhibition of oxidative stress and inflammation.

## 2 MATERIALS AND METHOD

### 2.1 Animals

C57 mice with age of eight weeks were provided by Experimental Animal Center of Ningxia Medical University, and calpain‐1 knockout (calpain‐1 KO) mice with age of eight weeks were purchased from Cyagen Biotechnology). The animals were maintained in the animal care facility at the Experimental Monitoring Laboratory of Ningxia Medical University at a temperature (~23°C) and humidity and were exposed to a 12/12‐hr light–dark cycle with ad libitum access to food and water. All experimental procedures were performed in accordance with the guidelines for the care and handling of laboratory animals recommended by the National Institutes of Health (NIH) and were approved by the Institutional Animal Care Committee (Protocol No 20,190,801). Standard diet (*SD*) and high‐fat diet (HFD, containing 25.4% of fat) for mice were purchased from Beijing Keao Xieli Feed Co., Ltd (Beijing, China). Mice were divided into four groups with 8 animals in each group: C57 + *SD*, calpain‐1 KO + *SD*, C57 + HFD, calpain‐1 KO + HFD. All mice were fed with *SD* or HFD for 16 weeks.

### 2.2 Body weight

At the start and end of the experiment, the body weights of animals were recorded.

### 2.3 Biochemical analysis

Serum levels of total cholesterol (TC), triglycerides (TG), low‐density lipoprotein cholesterol (LDL‐C), and high‐density lipoprotein cholesterol (HDL‐C), and activities of alanine aminotransferase (ALT) and aspartate aminotransferase (AST) were determined enzymatically by using commercial kits (Nanjing Jiancheng Biotechnology Company, Nanjing, China) according to the manufacturer's instructions. Total lipids were extracted from hepatic tissue using isopropanol, and contents of TC and TG were determined enzymatically by using commercial kits (Nanjing Jiancheng Biotechnology Company, Nanjing, China) according to the manufacturer's instructions.

### 2.4 Calpain activity

Lysates of liver tissue were used for measurement of the activity of calpain. Calpain activity was determined by using a fluorescence substrate N‐succinyl‐LLVY‐AMC (Amyjet Scientific Inc, Beijing, China) according to the manufacturer's instructions. The fluorescence intensity at 400 nm excitation and 505 nm emission wavelengths was measured using Synergy Multi‐Mode Microplate Reader (BioTek, Germany).

### 2.5 Contents of oxLDL, TNF‐α, and IL‐6

Determination of oxLDL, TNF‐α, and IL‐6 contents in serum and/or liver was performed using an ELISA kit following the manufacturer's instructions (Neobioscience, Shenzhen, China).

### 2.6 SOD activity and MDA content

SOD activity and MDA content in serum and liver were measured using commercial kits (Nanjing Jiancheng Biotechnology Company, Nanjing, China) according to the manufacturer's instructions.

### 2.7 Statistical analysis

Data are shown as the mean ± *SEM* and analyzed by one‐way analysis of variance (ANOVA) and Student's *t* test using SPSS 17.0 software. *p < .05 showed the statistically significant difference.

## 3 RESULTS

### 3.1 Effect of calpain‐1 on body weight and calpain activity in liver of mice fed with *SD* or HFD

**FIGURE 1:**
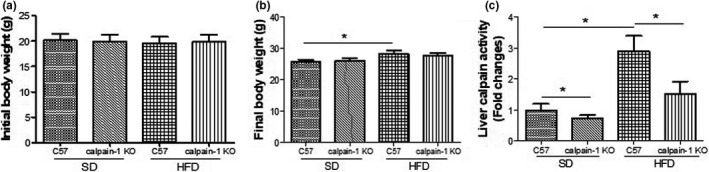
Effect of calpain‐1 knockout on body weight at the start (a) and the end of the experiment (b) and liver calpain activity (c) of mice fed with*SD*or HFD. Mice were divided into four groups: C57 + *SD*, calpain‐1 + *SD*, C57 + HFD, and calpain‐1 + HFD. All mice were fed with*SD*or HFD for 16 weeks. Data are expressed as means ± *SD*.*n* = 8. *p < .05 was considered statistically significant

As shown in Figure 1, the body weight at the start of the experiment showed no difference among four groups (Figure 1a). At the end of the experiment, the body weights of C57 and calpain‐1 KO mice both fed with *SD* are comparable (Figure 1b). However, C57 mice fed with HFD showed a significant increase in body weight compared with that of C57 mice fed with *SD*. No significant difference in body weight was found between C57 and calpain‐1 KO both fed with HFD. The calpain activity in liver was decreased in calpain‐1 KO mice compared with that in C57 mice both fed with *SD* (Figure 1c). Compared with that in C57 mice fed with *SD*, C57 mice fed with HFD showed a significant increase in liver calpain activity, which was significantly decreased in calpain‐1 KO mice fed with HFD.

### 3.2 Effect of calpain‐1 KO on activity of AST and ALT in serum of mice fed with *SD* or HFD

**FIGURE 2:**
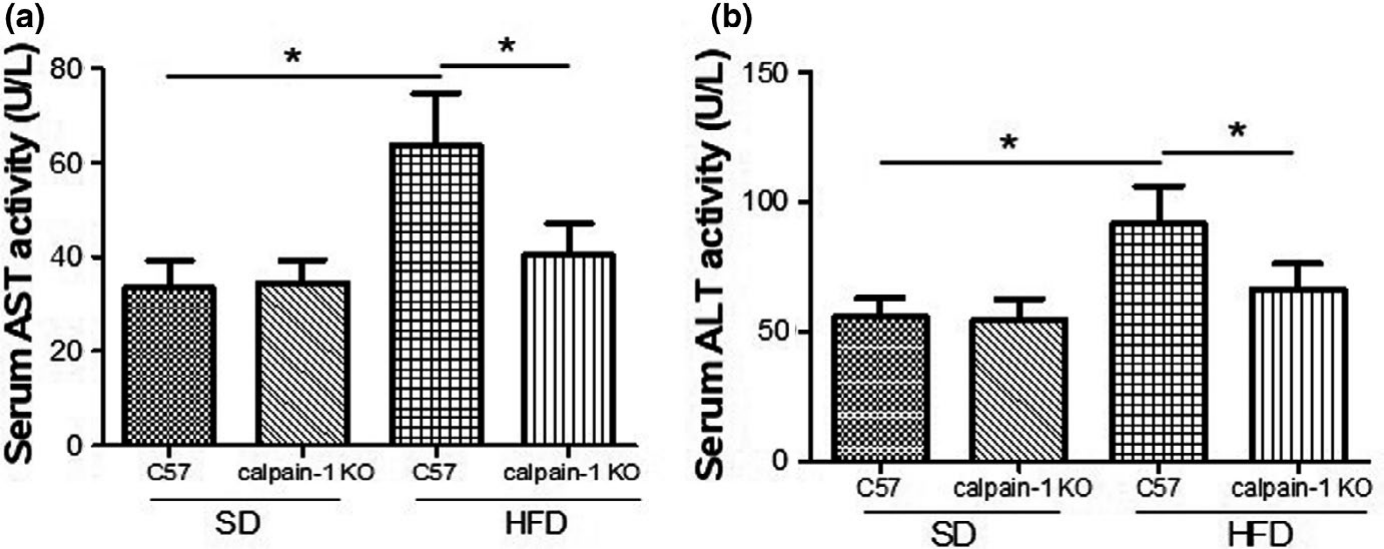
Effect of calpain‐1 knockout on AST (a) and ALT (b) of mice fed with*SD*or HFD. Mice were divided into four groups: C57 + *SD*, calpain‐1 + *SD*, C57 + HFD, and calpain‐1 + HFD. All mice were fed with*SD*or HFD for 16 weeks. Data are expressed as means ± *SD*.*n* = 8. *p < .05 was considered statistically significant

Serum activity of both AST and ALT of C57 mice fed with *SD* is comparable with that of calpain‐1 KO mice fed with *SD* (Figure 2a,b). However, compared with that of C57 mice fed with *SD*, C57 mice fed with HFD showed a significant increase in activity of both AST and ALT, which was significantly decreased in calpain‐1 KO mice fed with HFD. The results suggested that HFD induced the liver dysfunction, which was improved by knockout of calpain‐1.

### 3.3 Effect of calpain‐1 KO on lipid profiles in serum and liver of mice fed with *SD* or HFD

**FIGURE 3:**
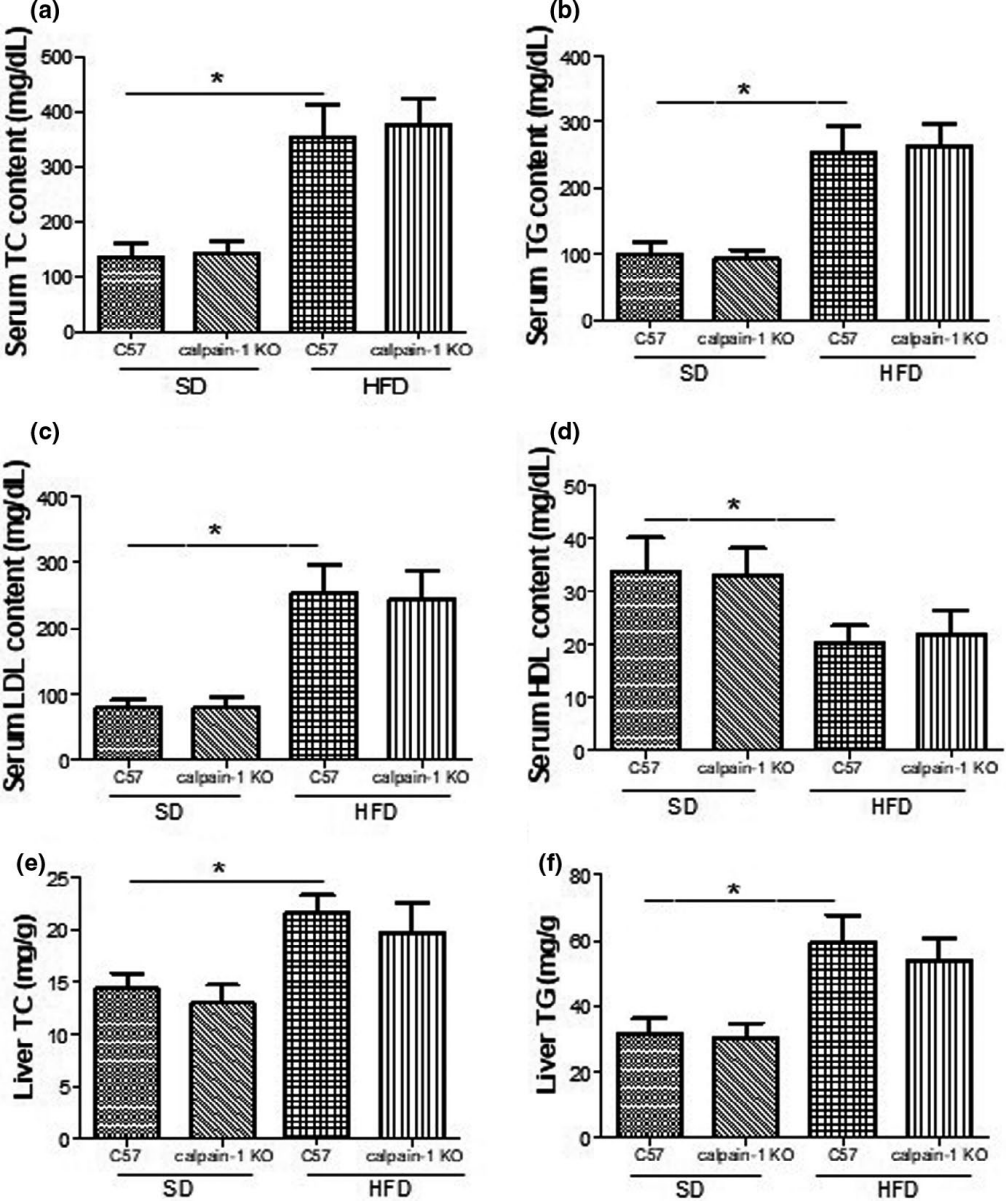
Effect of calpain‐1 knockout on contents of TC (a), TG (b), LDL (c), and HDL (d) in serum and contents of TC (e) and TG (f) in liver of mice fed with*SD*or HFD. Mice were divided into four groups: C57 + *SD*, calpain‐1 + *SD*, C57 + HFD, and calpain‐1 + HFD. All mice were fed with*SD*or HFD for 16 weeks. Data are expressed as means ± *SD*.*n* = 8. *p < .05 was considered statistically significant

Hyperlipidemia induces the lipid accumulation in liver, resulting in the liver dysfunction. To investigate the mechanism underlying the protection against liver injury by knockout of calpain‐1, the lipid profiles in serum were determined. As shown in Figure 3, the levels of TC (Figure 3a), TG (Figure 3b), LDL (Figure 3c), and HDL (Figure 3d) in serum were not found differently between C57 and calpain‐1 KO mice both fed with *SD*. However, compared with that of C57 mice fed with *SD*, C57 mice fed with HFD showed a significant increase in TC, TG, and LDL levels, and a decrease in HDL level. In addition, knockout of calpain‐1 had no effect on lipid profiles when both C57 and calpain‐1 mice were fed with HFD. Similar action pattern was found in terms of TC (Figure 3e) and TG (Figure 3f) contents in liver. The results suggested that knockout of calpain‐1 protects against liver dysfunction without affecting the lipid profiles in both serum and liver and that other mechanism might explain the protection.

### 3.4 Effect of calpain‐1 KO on contents of oxLDL and MDA and activity of SOD in serum or/and liver of mice fed with *SD* or HFD

**FIGURE 4:**
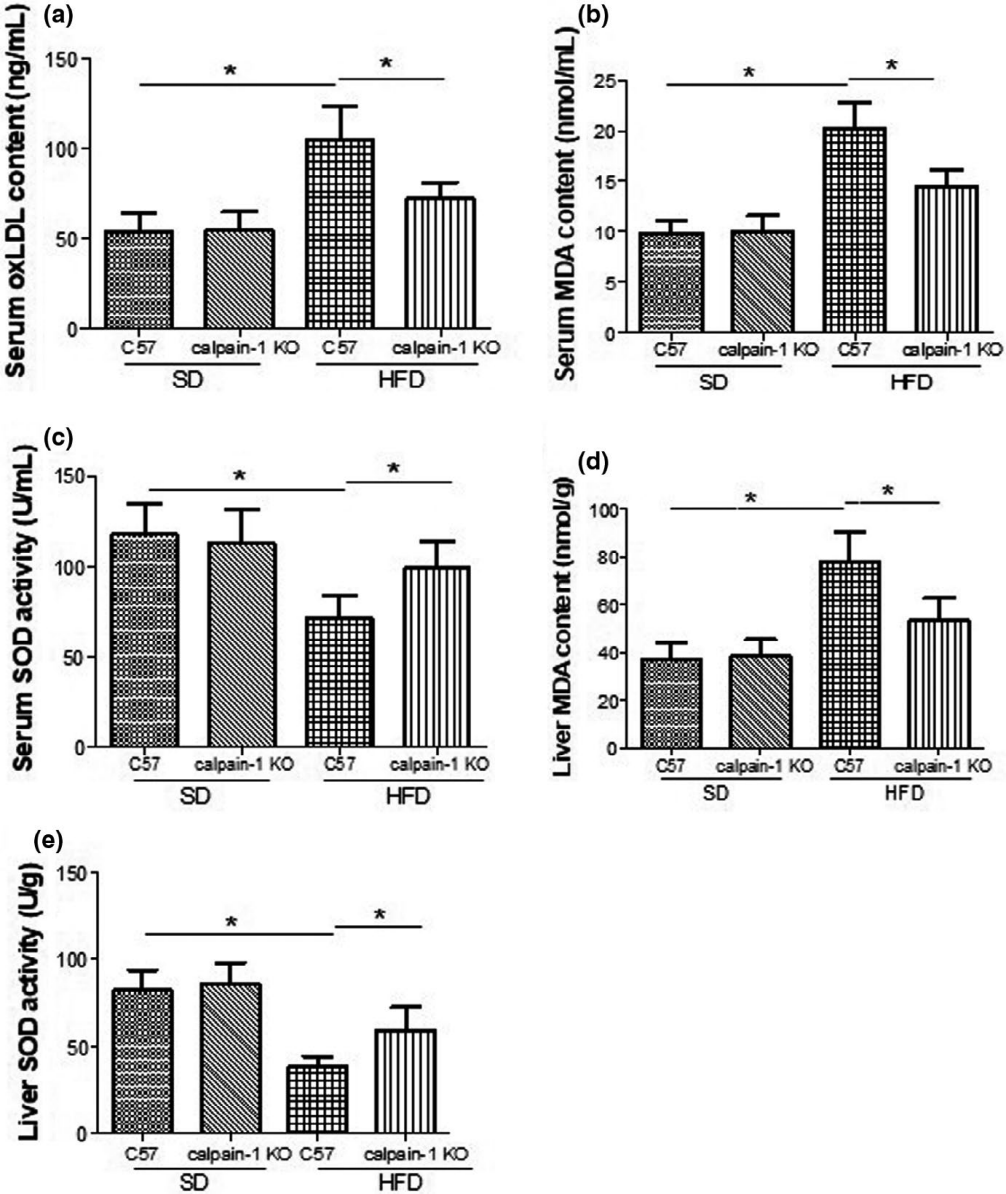
Effect of calpain‐1 knockout on contents of oxLDL (a), MDA (b), and SOD activity (c) in serum and contents of MDA (d) and SOD activity (e) in liver of mice fed with*SD*or HFD. Mice were divided into four groups: C57 + *SD*, calpain‐1 + *SD*, C57 + HFD, and calpain‐1 + HFD. All mice were fed with*SD*or HFD for 16 weeks. Data are expressed as means ± *SD*.*n* = 8. *p < .05 was considered statistically significant

Oxidation stress is implied in the pathogenesis of hyperlipidemia‐induced liver dysfunction. Imbalance between lipid peroxidation production and anti‐oxidation enzyme contributes to the oxidation stress. As shown in Figure 4, the contents of oxLDL (Figure 4a) and MDA (Figure 4b) and SOD activity (Figure 4c) in serum were not found differently between C57 and calpain‐1 KO mice both fed with *SD*. However, compared with that of C57 mice fed with *SD*, C57 mice fed with HFD showed a significant increase in oxLDL and MDA contents, and a decrease in SOD activity. In addition, knockout of calpain‐1 significantly decreased the contents of oxLDL and MDA, while increased the SOD activity in serum when both C57 and calpain‐1 mice were fed with HFD. Similar action pattern was found in terms of the MDA (Figure 4d) contents as well as the SOD activity (Figure 4e) in liver. The results suggested that the protection against liver dysfunction by knockout of calpain‐1 is at least partly attributed to the inhibition of oxidation stress.

### 3.5 Effect of calpain‐1 KO on TNF‐α and IL‐6 contents in serum and liver of mice fed with *SD* or HFD

**FIGURE 5:**
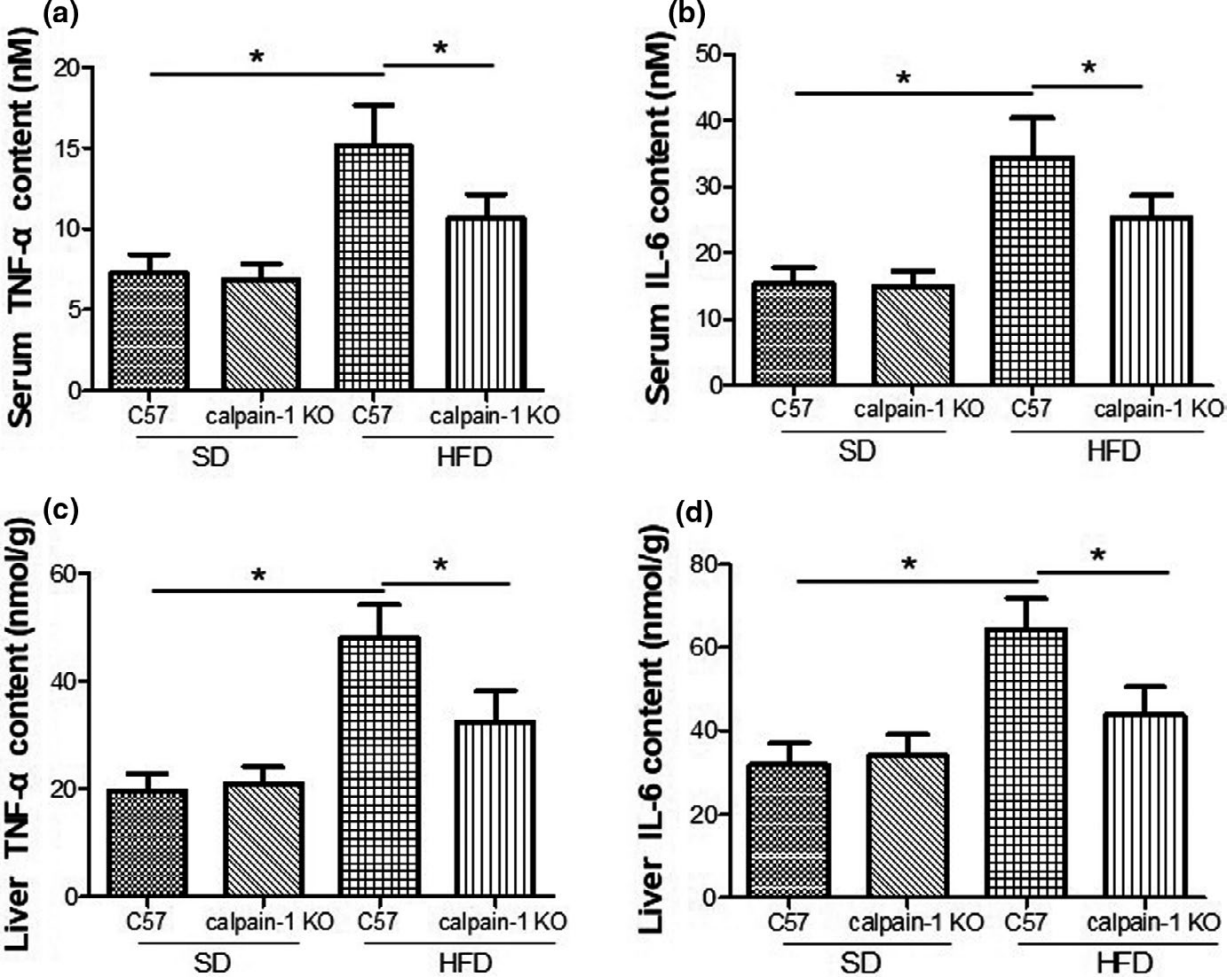
Effect of calpain‐1 knockout on TNF‐α (a, c) and IL‐6 (b, d) in mice fed with*SD*or HFD. Mice were divided into four groups: C57 + *SD*, calpain‐1 + *SD*, C57 + HFD, and calpain‐1 + HFD. All mice were fed with*SD*or HFD for 16 weeks. Data are expressed as means ± *SD*.*n* = 8. *p < .05 was considered statistically significant

Inflammation also contributes the pathogenesis of hyperlipidemia‐induced liver dysfunction. As shown in Figure 5, the contents of TNF‐α (Figure 5a,c) and IL‐6 (Figure 5b,d) in serum and liver were not found differently between C57 and calpain‐1 KO mice both fed with *SD*. However, compared with that of C57 mice fed with *SD*, C57 mice fed with HFD showed a significant increase in TNF‐α and IL‐6 contents in both serum and liver. In addition, knockout of calpain‐1 significantly decreased the contents of TNF‐α and IL‐6 when both C57 and calpain‐1 mice were fed with HFD, suggesting that anti‐inflammation might be another essential mechanism by which knockout of calpain‐1 protected against liver dysfunction.

## 4 DISCUSSION

The present study showed that knockout of calpain‐1 decreased activities of calpain, AST, and ALT, reduced the contents of oxLDL, MDA, TNF‐α, and IL‐6, while increased the SOD activity in serum and/or liver mice fed with HFD. The results suggested that knockout of calpain‐1 protected against hyperlipidemia‐induced liver dysfunction through inhibition of oxidative stress and inflammation.

The results that knockout of calpain‐1 reduced serum activity of both AST and ALT showed that calpain‐1 is involved in the hyperlipidemia‐induced liver dysfunction. Consistently, it has been previously reported that calpain‐1 is upregulated in the liver tissue of mice fed with HFD, highlighting the important role of calpain‐1 in the hyperlipidemia‐induced liver dysfunction (Lee et al., 2019). The involvement of calpain‐1 in liver dysfunction largely depends on its mediation of oxidative stress and inflammation, which are the most important contributors to the onset and progression of liver dysfunction (Li & Lu, 2018).

Hyperlipidemia induces the lipid accumulation in liver, resulting in the liver dysfunction (Qi et al., 2019; Ramos et al., 2020; Saadati et al., 2019). To investigate the mechanism underlying the protection against liver dysfunction by knockout of calpain‐1, the lipid profiles in serum and liver were determined. The results showed that HFD increased the levels of TC, TG, and LDL, while decreased HDL content in serum and/or liver. However, knockout of calpain‐1 had no effect on lipid profiles. The results suggested that knockout of calpain‐1 protected against liver injury without affecting the lipid profiles in both serum and liver and that other mechanisms might explain the attenuation.

Oxidation stress is implied in the pathogenesis of hyperlipidemia‐induced liver dysfunction (Kang et al., 2014; Tanwar et al., 2020; Tilg & Moschen, 2008). Imbalance between lipid peroxidation production and anti‐oxidation enzyme contributes to the oxidation stress. The present study showed that knockout of calpain‐1 significantly decreased the contents of oxLDL and MDA, while increased the SOD activity when both C57 and calpain‐1 mice were fed with HFD. Similarly, several studies reported the involvement of calpain‐1 in oxidative stress (Tucker et al., 2020; Wang et al., 2001; Xia et al., 2013). For example, astragaloside IV was found to protect against hyperglycemia‐induced vascular endothelial dysfunction by inhibiting oxidative stress and calpain‐1 activation (Wang et al., 2001). In addition, targeted gene inactivation of calpain‐1 suppresses cortical degeneration due to traumatic brain injury and neuronal apoptosis induced by oxidative stress (Tucker et al., 2020). Furthermore, simvastatin improves cardiac hypertrophy in diabetic rats by attenuation of oxidative stress and inflammation induced by calpain‐1 activation. These results suggested that protection against liver dysfunction by knockout of calpain‐1 is at least partly attributed to the inhibition of oxidation stress.

Inflammation also contributes the pathogenesis of hyperlipidemia‐induced liver injury and the related disorders (Yamada et al., 2012; Yin et al., 2017; Yu et al., 2019). Hyperlipidemia‐induced fat accumulation in liver has been linked with insulin resistance, obesity, and metabolic syndrome (Jiang et al., 2018). Visceral adipose tissue plays a significant role in obesity pathophysiology (Jiang et al., 2018). The present study demonstrated that knockout of calpain‐1 significantly decreased the contents of TNF‐α and IL‐6 when both C57 and calpain‐1 mice were fed with HFD, suggesting that anti‐inflammation might be another essential mechanism by which knockout of calpain‐1 improved the liver dysfunction. Similarly, several studies reported the involvement of calpain‐1 in apoptosis and inflammation (Xia et al., 2013; Yuan et al., 2016; Zhang et al., 2011). Cytoprotective effect of prednisolone in hepatic IR injury was closely associated with suppression of IL‐beta/TNF‐alpha production and calpain‐1 activation (Zhang et al., 2011). Calpain released from dying hepatocytes mediates progression of acute liver injury induced by model hepatotoxicants (Yuan et al., 2016). Nicotinic acetylcholine receptor alpha1 was found to promote calpain‐1 activation and macrophage inflammation in hypercholesterolemic nephropathy (Zhang et al., 2020). In the previous study, we found that calpain‐1 plays important role in inflammation and the related cardiovascular diseases such as atherosclerosis and cardiac hypertrophy (Xia et al., 2013; Zhang et al., 2019).

In summary, calpain‐1 plays an essential role in pathogenesis of hyperlipidemia‐induced liver dysfunction. A better understanding of the pathophysiological processes underlying liver injury will allow the development of new therapeutic approaches targeting calpain‐1.

## ETHICAL REVIEW

All experimental procedures were performed in accordance with the guidelines for the care and handling of laboratory animals recommended by the National Institutes of Health (NIH), and the protocol was approved by the Ethics Committee of Ningxia Medical University, China.

## CONFLICT OF INTEREST

The authors of this manuscript state that they do not have conflict of interest to declare.

## Data Availability

The data that support the findings of this study are available from the corresponding author upon reasonable request.
